# Supplementary material for: MetaRibo-Seq measures translation in microbiomes
Source: Nat Commun. 2020 Jun 29;11:3268. doi: 10.1038/s41467-020-17081-z (PMC7324362; doi:10.1038/s41467-020-17081-z)
Supplement: Supplementary file 10 — Supplementary Data 7 [file 41467_2020_17081_MOESM10_ESM.zip › File2/Confidence_High_Taxonomy/71145_out.krona.html]

Javascript must be enabled to view this page.

members
magnitude
magnitudeUnassigned
count
unassigned
taxon
rank

71145\_out

2

2
superkingdom
2

2
1239
phylum

2
class
186801

186802
order
2

186803
family
2

2
1506553
genus

2
species
333367

SRS065397\_contig\_number\_10610SRS146813\_contig\_number\_contig-100\_1067.148338
